# A pseudo-randomised clinical trial of *in *situ gels of fluconazole for the treatment of oropharngeal candidiasis

**DOI:** 10.1186/1745-6215-12-99

**Published:** 2011-04-19

**Authors:** Harish M Nairy, Narayana R Charyulu, Veena A Shetty, Prabhu Prabhakara

**Affiliations:** 1 Department of Pharmaceutics, NGSM Institute of Pharmaceutical Sciences, Paneer, Deralakatte Post, Mangalore, India; 2 Department of Microbiology, KS Hegde Medical Academy, Deralakatte, Mangalore, India

## Abstract

**Background:**

Oropharyngeal candidasis is a common opportunistic infection seen in immunocompromised patients. Fluconazole has a broad spectrum antifungal activity including a wide variety of *candida *species. Aim of the present investigation was to formulate and find out the relative efficacy of *in situ *gels of fluconazole.

**Method:**

The *in situ *gels were prepared using polymers which exhibited sol-to-gel phase transition due to change in specific physico-chemical parameters, such as ion triggered system using gellan gum (0.5% w/v) along with sodium carboxylmethylcellulose (0.35%w/v). The study design was bicenter, 'pseudo-randomised, single blind trial conducted in Mangalore., India, which includes 15 HIV positive patients, 15 patients with partial or completes dentures, and 15 patients who were treated with (active control) fluconazole tablets 100 mg/day for 14 days. Severity of disease was scored clinically before treatment and at clinical evaluations on day 3, 7, 14, 18, 21, 35, and 42. Semiquantitative microbiological cultures of oral swabs were also obtained on same days.

**Results:**

All patients had mycological documented oropharyngeal candidiasis and were treated with fluconazole (0.5%w/v) *in situ *gels for 14 days Severity of disease was scored clinically before treatment and at different predetermined time intervals along with semi quantitative culture of oral swabs. The clinical response rate showed 97% cure after 14 days in the treated with *in situ *gel. In comparison, the control group treated with fluconazole tablets showed 85% improvement in symptoms of oral candidiasis. The patients suffering from HIV infection showed relapse in oral candidiasis at the end of 21 days. The patients having oral candidiasis due to partial or complete dentures showed complete recovery and were free from signs and symptoms of oral candidiasis.

**Conclusions:**

The *in situ *gel formulation of fluconazole was well tolerated with no severe adverse reaction and offers a better alternative to tablet formulation in the treatment of oropharyngeal candidasis.

**Trial registration:**

Current Controlled Trails ISRCTN90634047

## Background

Fungal pathogens increasingly cause nosocomial infections, especially among the surgical patients and the high risk critically ill, with an attributable death rate estimated at 38% [1]. Local delivery of drugs to the tissues of the oral cavity has a number of applications including the treatment of toothache, periodontal diseases, dental caries, bacterial and fungal infections. The conventional formulations for the local delivery of drugs to the oral cavity are the mouth paints, rinses, troches, creams, oral tablets and suspensions. But limitations include, poor bioavailability, poor availability of drug at the site of action and patient compliance. One way to improve the efficacy of the dosage form is to deliver the antifungal agent locally in the oral cavity [2,3] on the effected site. Better stability and longer residence time of locally administered antifungal agent will allow better penetration through the oral mucosal layer to act on *Candida *species.

*In situ g*els can resist the physiological stress caused by the skin flexion, mastication and movement of tongue, adopting shape of the applied area and controlling the drug release for prolonged time. Fluconazole is an orally active bistriazole antifungal agent which is used in the treatment of superficial and systemic candidasis [4]. The present dosage regimen of fluconazole for the treatment of oropharyngeal candidasis (OPC) of 100 mg tablets of fluconazole tablet twice daily. The occurrence of OPC in patients deteriorates the oral mucosa; increases pain, dysphagia, and anorexia; alters taste; and contributes toward worsening nutritional and general status. Recurrence of infection is usually seen in approximately 33% of patients treated with tablet formulation of fluconazole. Thus, there is a need for a topical agent which is as effective as systemic agent upon single administration, well tolerated and would not increase the risks of emergence of resistance [5]. Rene-jean Bensadown et al., demonstrated the ability to deliver high and prolonged salivary concentration of 50 mg mucoadhesive buccal tablets of miconazole helped in reduction of dose, reduced the risk of systemic exposure, drug-drug interaction and other toxicity [6].

In view of eliminating the signs and symptoms of OPC, present study was focused on formulation of *in situ *gels of fluconazole and evaluated for its physico-chemical properties as well as *in vivo *efficacy in patients.

## Objectives

The primary objective of study was to assess efficacy of topical application of *in situ *gels of fluconazole for the treatment of oropharyngeal candidiasis. The secondary objective of this study was to assess the microbiological colony count from the baseline to the end of the follow up term.

## Methods

### Materials

Fluconazole was a gift sample from M/s Dee Pharma Ltd., New Delhi, India. Gellan gum was procured from Hi-Media Pvt. Ltd, Mumbai, India. All other materials were used of analytical grade. Sub cultures of *Candida Spp*. were obtained from MTCC (Microbial Type Culture Collection), Chandigarh, India.

### Preparation of in situ gelling system

Gellan gum solutions of 0.5%w/v was prepared by adding the gum to deionized water containing 0.17%w/v sodium citrate and heated up to 90°C with continuous stirring. After cooling below 40°C an appropriate amount of calcium chloride (0.05%w/v) was added into the sol. Fluconazole (0.5%w/v) was dissolved in water and added to the above solution. The mixture was shaken using magnetic stirrer under aseptic condition to ensure thorough mixing. The composition of formulation under study is shown in Table 1. The formulation and characterization of the *in situ *gels has earlier reported [7]. The concentration of 0.5%w/v of fluconazole was taken based on Minimum inhibitory concentration (MIC) to show antifungal activity.

**Table 1:** *In vitro *characterization of *in situ *formulation

Formu-ation	pH*	Viscosity* (cps)	Spreadibility g.cm/s	Drug Content (%w/w)	*Muco-adhesive force dynes/cm^2^	Gelling Capacity	Gel strength g/s
F1	7.2	14000	28.5	90.4	52.5 ± 7.5	+++	6.5

* Average of three reading

- No gelation; + Gel after few minutes, dissolved rapidly; ++ Gelation immediately, remains for few hours; +++ Gelation immediately, remains for extended period.

#### Characterization of the *in situ *gels

The prepared gels were subjected for different parameters such as gelling capacity, viscosity measurement of both sols as well as corresponding gels, gel strength, mucoadhesive force, spreadability *in situ*, release studies, *in vitro *antifungal studies and *in vivo *evaluation.[7,8]

### In situ release studies

*In situ *release studies were carried out using, a "flow through cell" Dissolution medium pH 6.8 (simulated salivary pH) was pumped at a flow rate of 0.6 ml/min (corresponding to mean resting salivary flow rate) using flow regulators. Sols were added from the top, so that upon contact with the simulated salivary fluid the transition took place from sol to gel. The gels settled on the mucous membrane with mucoadhesion. Samples of 2 ml were withdrawn at different time intervals from the reservoir till the gel completely eroded. The cumulative percent drug released was determined by measuring the absorbance at 260 nm.

### In Vitro antifungal studies

The antifungal efficacy onof *in situ *gel formulation on *Candida *Spp. was determined by agar diffusion method employing 'cup plate technique'. Sterile solutions of fluconazole in water and the developed gel having the pH adjusted to 7.0, were poured into cups (0.1 ml of 0.1%w/v) bored into sterile malt yeast agar previously seeded with test organism. After allowing diffusion of the solutions for 2 h, the agar plates were incubated at 37°C for 24 h. The zone of inhibition (ZOI) was measured around each cup was compared with that of pure drug. The entire operation was carried out under aseptic condition in triplicate. Both positive and negative controls were maintained during the study [9].

### In vivo studies

#### Selection criteria of Patients

##### A. Inclusion criteria

. Patients of either sex (age group of 18 or above), with HIV-antibody seropositive or with partial or complete denture and the clinical picture of oropharyngeal candidiasis, which is characterized by creamy, white, curd like patches, removable erythematous lesions on the oral mucosal surfaces. On direct microscopic examination of samples of these infectious patches must reveal the presence of *candida *Spp., consistently and further must get confirmed the presence of mycological culture and differentiation of strains using Hicrome^® ^medium is used.

##### B. Exclusion criteria

A history of significant hepatic abnormalities or hepatic diseases and a life expectancy of less than 1 month or a clinical condition such that study completion could not be assured. Patients with a history of hypersensitivity to imidazole or azole compound and also patient who required therapy with other antifungal agents, H_2_-receptor blockers, antacids, rifampicin, phenobarbital, pheytoin, carbamazeme, terfenadine, or astimazole. Pregnant or lactating women were also excluded from the study.

The study protocol was approved by institutional review board and each patient signed a written statement of informed consent prior to receiving the study medication. The clinical study protocol was approved by the Institutional Human Ethics Committee (Approval No: ABSM/EC/18/2008)

### Study design

A bicenter, open-label, single blind, pseudo-randomised clinical trial design was followed, to compare the efficacy of fluconazole *in situ *gel (0.5% w/v) with that of fluconazole tablets (100 mg) for 14 days. The patients with OPC were divided into 3 groups, each comprising of 15 patients. Group I comprised of patients having HIV/AIDS treated with *in situ *gel, Group II comprised of patients with partial or complete dentures, treated with *in situ *gel and group III were comprised of patients with HIV/AIDS treated with fluconazole tablets 100 mg for 14 days. The patients demographic and baseline characters are shown in table 2.

**Table 2:** Patient demographic and base line characteristics

Variable	Treatment group			Total
	**Fluconazole treated group with HIV**	**Fluconazole treated group with partial or complete dentures**	**Active control**	

Gender, number				
Male	10	8	7	25
Female	05	5	8	20
Age				
Mean ± S.D	32 ± 3.5	45 ± 4.5	35 ± 1.5	
CD_4 _Count/mm^3^ Mean ± S.D	172 ± 78	650 ± 150	200 ± 250	
Pre treatment culture				
*Candida albicans*	12	10	13	
*Candida Parapsilosis*	-	01	-	
*Candida Dubliniansis*	03	04	02	

Following the initial (base line) visit, the subsequent visits were scheduled on day 3, 7, 14, 18, 22, 34, and 44. The efficacy of the dosage form was compared with tablets for the successful clinical response and changes from baseline for the symptoms of OPC, which included the soreness erythema, extent of oral lesions and quantification of colony forming units (CFU) of *Candida *spp. Clinical assessment were made and recorded by a Registered clinical practitioner. The severity of baseline, symptoms was assessed on a scale of 0 to 3 (0 = absent, 1 = mild, 2 = moderate, 3 = severe). The baseline severity of signs and symptoms of OPC are given in table 3. The quantification of CFU of *Candida spp*., was done by taking oral swabs at three different locations. Oral swabs were then streaked on Hichrome^® ^medium. The specimen was examined microscopically for hyphae, color of colony to distinguish the strain.

**Table 3:** Comparison of disease severity at baseline and at the end of the study period

Parameter	Group I	Group II	Group III	Group I	Group II	Group III	P Value
		
	At day 0 (Baseline)			At day 44 (end of study period)			
Extent of Lesion	1.03 ± 0.01	0.2 ± 0.02	1.13 ± 0.01	0.94 ± 0.02	0.01 ± 0.00	1.11 ± 0.01	0.36

Soreness/Burning	0.36 ± 0.05	0.13 ± 0.06	0.56 ± 0.05	0.46 ± 0.05	0.03 ± 0.00	0.66 ± 0.05	0.39

Erythema	0.93 ± 0.07	0.35 ± 0.01	1.03 ± 0.07	0.96 ± 0.05	0.00 ± 0.00	0.98 ± 0.07	0.42

### Clinical efficacy evaluation

The primary efficacy parameter for clinical response was rated according to the change in signs and symptoms from the baseline. The assessment was rated as "cured" (clearance of all signs and symptoms), "improved" (minimal signs and symptoms with no residual visible candida lesions), unchanged (no change in signs and symptoms), or "deteriorated" (worsening or increasing signs and symptoms). Patients with a clinical evaluation of "cured" or "improved" were considered as a successful outcome of the study.

Secondary efficacy parameters included the quantification in terms of CFUs of *Candida *spp. *a*nd results of culture from swabs taken at the same sites used at baseline. A mycological cure was defined as a yeast quantification of <10 CFU/ml based on comparison of mycological culture from swabs collected from healthy volunteers.

To analyze the data of clinical efficacy statistically, a two-factor repeated measures analysis of variance was used between the treated groups over a period of 44 days. The criterion for significance was set at p < 0.05.

## Results

Sol to gel transformation of gellan occurs in the presence of either monovalent or divalent cations in contact with the salivary fluids. The quantities of the complexing agents calcium chloride and sodium citrate must be such that there is no free calcium in ionic form in the formulation so as to ensure that they are in fluid state before administration, but sufficient Ca^++ ^ions must be released when the complex is broken down (due to dissociation) in presence of simulated salivary fluid to cause gelation. It was reported earlier that optimum concentration of calcium chloride (0.05%w/v) with 0.17% w/v sodium citrate is used along with gellan gum (0.5%w/v) were found to be satisfactory to cause gelation and gelling capacity. Aqueous sols exhibited pH value of 7.2 at 25°C (Table 1). The formulation exhibited shear thinning pseudo-plastic behavior with thixotrophy. Further, it was also found that, the formulations were liquid at room temperature (25°C) with viscosity of 85 cps which underwent rapid gelation when the pH was raised to 6.8 which contributed to the increased viscosity of 14000 cps. The mucoadhesive force is an important physicochemical parameter for topical application in buccal cavity which was found to be 55 dynes/cm^2^. The values are shown in table 1

### *In vitro *drug release

The *in vitro *release of drug from these gels was characterized by an initial phase of high release (burst effect). However, as gelation proceeds, the remaining drug was released at a slower rate followed by a second phase of moderate release. The formulations showed sustained release up to 8 h. At the end of 8 h 98% drug was released from the formulation.

### In vivo evaluation

A total of 45 patients which included 15 patients who were HIV positive, 15 patients with partial or complete dentures, and the third group consisted of 15 patients with HIV positive patients treated with fluconazole tablets. All patients were confirmed of oropharyngeal candidiasis.. A 5 ml of the dosage form was administered every 12 h for a period of 14 days at the infected area using syringe. The dosage form upon contact with saliva formed gel (within >4 s) and was visible for a period of 6 to 8 h. At the end of the treatment phase, the clinical response rate was assessed. From group I, 12 out of 15 patients (80%) showed good clinical response up to a period of 21 days. After which the relapse were seen in 90% cases having HIV. It was considered as "cured" for a period of 21 days for this group. Whereas the group II, the clinical efficacy "cured" were seen in 15 out of 15 patients (100%) and relapse were not seen up to 44 days. It was considered as "cured" for group II for a period of 44 days (study duration). In group III, 10 out of 15 patients (66.6%) were "cured" at the end of 14^th ^day, whereas at the end of 18^th ^day showed relapse in 8 out of 10 (80%) "cured" cases. The other patients did not show any clinical response and no change from baseline.

**Figure 1:**
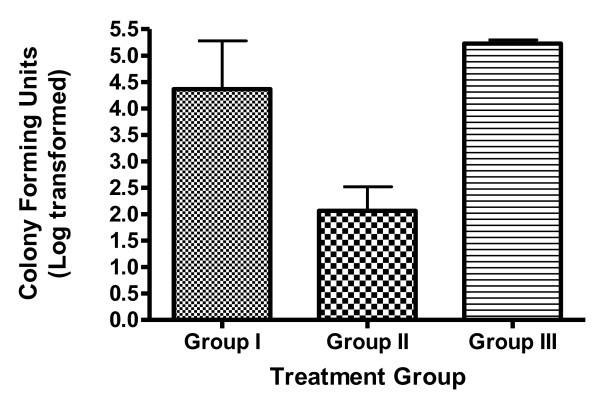
**Comparative microbiological studies in terms of colony forming units for oral swabs at the end of 44 days**.

The microbiological investigation at baseline, all the treated groups showed positive microscopic result for OPC. At day 14, that is at the end of the treatment the percentage of subjects with a negative microscopic examination increased to approximately 85%, 95% and 75% for group I, II and III respectively from the baseline. During the follow-up period, the microbiological investigation was done to understand the relapse in OPC, which showed that at the end of 44^th ^day the group I has an increase in positive microscopic results (100%), whereas in group II no changes and in group III there was increase in CFUs (300/ml) in 100% cases which showed negative at the end of 14^th ^day, indicating relapse thereafter. (Figure 1). The most common species isolated was *Candida albicans *(30 out of 45 cases) strains.

## Discussion

Oral candidiasis threatens all kinds of immunocompromised patients and may recur repeatedly during the course of their underlying disease. Topical agents generally have been used in the early stages, where as systemic therapy is limited to patients with poor tolerance. In the present investigation *in situ *gels of fluconazole was formulated and evaluated for the *in vitro *and *in vivo *characteristics. It is established that formulations containing calcium carbonate produce a significantly produced stronger gel than those containing sodium bicarbonate. This is due to the internal ionotropic gelation effect of calcium on gellan gum [7,8]. The present investigation utilized Ca^++ ^ions in complexed form with sodium citrate (0.17%w/v) which upon contact with the saliva formed gel which would release the drug for a longer duration of time. The slightly acidic conditions of the buccal cavity ensured reproducible gelation of the gellan gum. Low level of cations present in the solution was sufficient to hold the molecular chains together and inhibit hydration. A reduction in the concentration of gellan gum without compromising the gelling capacity and rheological properties of the delivery system may be achieved by the addition of viscosity enhancing polymers such as sodium carboxylmethylcellulose (NaCMC). Since NaCMC helped the gel to rapidly settle because of its higher density, the gels showed better adhesion property to the mucous membrane and subsequently prolonged release. The formulation showed optimum pH of 7.0 ± 0.3 which is very essential to reduce irritation. The formulation showed good sol to gel conversion with low viscosity of the sols and subsequent gels showed higher viscosity. The higher viscosity could contribute in better mucoadhesive property and retentive property. The *in vitro *release profile was conducted for a period of 6 h and showed biphasic release pattern which is a characteristic feature of matrix diffusion kinetics. The initial burst effect was considerably reduced with increase in polymer concentration.

**Figure 2:**
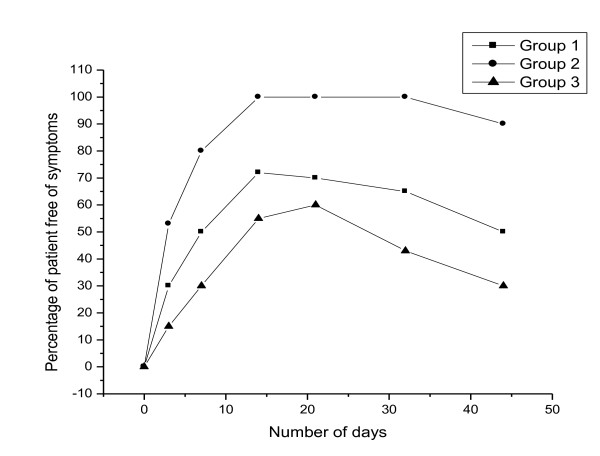
**Percentage of patients who were free from symptoms of candidal infection**.

*In vivo *evaluation showed that all the three groups showed good clinical response during the treatment duration (14 days). Group I, group II and group III showed clinically cured for 95%, 98% and 93% of the patients respectively. During the follow up the patients treated with fluconazole tablets showed higher incidence of relapse at faster rate (within 18 days). The group II patients did not show any signs of relapse for a period of 44 days. No statistically significant differences between different treated groups were observed in the clinical response during the treatment period. The faster relapse were seen in patients treated with oral tablets was may be due to lower drug concentration at the site of infection. *C. albicans *was cultured in more than 66.6% in all treated groups. Because of immunosupression in HIV patients, >90% of the cases showed relapses at the end of 21 days and remained "unchanged" which was same in case of group III, Significant clinical efficacy was seen in patients of group II with no relapse and the patients were clinically "cured". A comparison of various treated groups of patients free from OPC is given in Figure 2. Bonferroni's Multiple Comparison Test studies showed no significant difference in clinical response (P < 0.005) between the treated groups at the end of 14 days. But the significance did not persist after 22 days between treated groups. Microbiological investigation revealed a statistically significant difference (P > 0.001) when the different treated groups were compared for a period of 14 days. *In situ *gels were more effective than fluconazole tablets in producing greater rates of negative culture and better clinical response. The more conventional method of assessing mycological response-negative culture demonstrated a significant difference in the ability of *In situ *gels to eradicate *Candida *species at the end of therapy when compared with fluconazole tablets. The results of the *in vivo *evaluation demonstrated that fluconazole *in situ *gels for 14 days (0.5%w/v) was effective in treating of oral candidiasis with patients suffering from HIV/AIDS and with partial or complete dentures. *In situ *gels were well tolerated by the patients in the trails. Adverse events reported in the literature tend to be relatively minor and usually related to the gastrointestinal tract [10,11]. The finding was confirmed in our study as well. Gastrointestinal symptoms (eg, nausea, diarrhea, abdominal pain) were experienced by approximately 6 patients in the treated groups.

## Conclusion

In conclusion, the *in situ *gel formulation of fluconazole has shown significant clinical efficacy in the treatment of oropharyngeal candidiasis and produced successful clinical outcomes. The *in situ *gels were easier to administer, well tolerated and showed better clinical efficacy in treatment of oropharyngeal candidiasis in patients with partial or complete dentures or HIV/AIDS patient and can be considered as a viable alternative to the current conventional formulations available for the treatment of candidiasis.

## Competing interests

The authors declare that they have no competing interests.

## Authors' contributions

HMN is the trial co-ordinator. RNA made substantial contributions to the conception and design of the study, and is co-responsible for the overall direction of the project, the analysis and interpretation of data. VS is co-responsible for the overall design, administration and direction of the study. PP also participated in the design and direction of the study. All authors have read and approved the final version.
